# Biobutanol production in a *Clostridium acetobutylicum* biofilm reactor integrated with simultaneous product recovery by adsorption

**DOI:** 10.1186/1754-6834-7-5

**Published:** 2014-01-08

**Authors:** Dong Liu, Yong Chen, Feng-Ying Ding, Ting Zhao, Jing-Lan Wu, Ting Guo, Heng-Fei Ren, Bing-Bing Li, Huan-Qing Niu, Zhi Cao, Xiao-Qing Lin, Jing-Jing Xie, Xue-Jun He, Han-Jie Ying

**Affiliations:** 1 State Key Laboratory of Materials-Oriented Chemical Engineering, College of Biotechnology and Pharmaceutical Engineering, Nanjing University of Technology, No. 30, Puzhu South Road, Nanjing 211816, China

**Keywords:** Biofilm reactor, *Clostridium acetobutylicum*, Simultaneous product recovery, Acetoin, Adsorption, Redox modulation

## Abstract

**Background:**

*Clostridium acetobutylicum* can propagate on fibrous matrices and form biofilms that have improved butanol tolerance and a high fermentation rate and can be repeatedly used. Previously, a novel macroporous resin, KA-I, was synthesized in our laboratory and was demonstrated to be a good adsorbent with high selectivity and capacity for butanol recovery from a model solution. Based on these results, we aimed to develop a process integrating a biofilm reactor with simultaneous product recovery using the KA-I resin to maximize the production efficiency of biobutanol.

**Results:**

KA-I showed great affinity for butanol and butyrate and could selectively enhance acetoin production at the expense of acetone during the fermentation. The biofilm reactor exhibited high productivity with considerably low broth turbidity during repeated batch fermentations. By maintaining the butanol level above 6.5 g/L in the biofilm reactor, butyrate adsorption by the KA-I resin was effectively reduced. Co-adsorption of acetone by the resin improved the fermentation performance. By redox modulation with methyl viologen (MV), the butanol-acetone ratio and the total product yield increased. An equivalent solvent titer of 96.5 to 130.7 g/L was achieved with a productivity of 1.0 to 1.5 g · L^-1^ · h^-1^. The solvent concentration and productivity increased by 4 to 6-fold and 3 to 5-fold, respectively, compared to traditional batch fermentation using planktonic culture.

**Conclusions:**

Compared to the conventional process, the integrated process dramatically improved the productivity and reduced the energy consumption as well as water usage in biobutanol production. While genetic engineering focuses on strain improvement to enhance butanol production, process development can fully exploit the productivity of a strain and maximize the production efficiency.

## Background

Butanol fermentation, commonly known as ABE (acetone, butanol, and ethanol) fermentation, once the second largest biotechnological industry in the world [1], has attracted renewed interest in recent years for several economic and environmental reasons [2,3]. However, low alcohol yield (<25% w/w), low reactor productivity (approximately 0.3 g · L^-1^ · h^-1^), and high energy-consumption and water usage due to low solvent titer (approximately 20 g/L) are major challenges of conventional ABE fermentation, limiting the development of economically viable biobutanol production [2,3].

The low butanol yield in ABE fermentation is largely attributed to the fact that considerable amounts of glucose are converted to acetone with concomitant release of CO_2_ and H_2_. Genetic engineering technologies, for example, disrupting the *adc* gene [4] or reinforcing the direct butanol-forming route [5], have been applied to improve butanol yield. Introducing CO_2_ fixation pathways into a butanol-producing host may also be a promising approach to improve the product yield [6]. To address the low solvent-productivity, immobilized cell systems have been developed that maintain a high cell-activity and reduce the downtime of a repeated batch fermentation mode or continuous fermentation mode [7]. The highest ABE productivity, 15.8 g · L^-1^ · h^-1^ was obtained by continuous fermentation of immobilized *Clostridium beijerinckii* (*C. beijerinckii*) cells adsorbed to clay brick; this productivity was approximately 50 times the typical productivity of planktonic cell fermentation [8]. However, the butanol titer in this process was only about 5 g/L. A more elaborate process should be designed to avoid the loss of substrate in the effluent and to increase the solvent titer.

Product toxicity to the producing strain at low solvent-concentrations is another critical limitation of biobutanol production. Due to the susceptibility of *Clostridia* to butanol, the solvent titer during ABE fermentation is low. Until now, the highest butanol concentration achieved in the fermentation broth without simultaneous product recovery was only 20.9 g/L, which was obtained in a batch fermentation using *C. beijerinckii* BA101 generated by nitrosoguanidine (NTG) mutagenesis [9]. Solvent recovery using conventional distillation is energy intensive and expensive. Low butanol titers increase recovery costs and water usage. Numerous processes have been developed for simultaneous recovery of solvent from the broth during fermentation. The most common solvent recovery techniques are liquid-liquid extraction, gas stripping, pervaporation, and adsorption. In particular, gas stripping has been extensively explored in recent years, and attractive results have been obtained in terms of solvent concentration and productivity. By fed-batch culture of a genetically engineered strain with gas-stripping recovery, 585.3 g of butanol was produced from 1,861.9 g of glucose, with a productivity of 1.32 g · L^-1^ · h^-1^[5]. Similarly, by applying gas stripping intermittently in fed-batch fermentation with a butanol tolerant strain, 172 g/L ABE (containing 113.3 g/L butanol) were produced with an overall ABE productivity of 0.53 g · L^-1^ · h^-1^[10]. Although gas stripping is a favorable choice due to its operation simplicity, it usually requires a higher energy-input because of the intensive product-capturing step [11]. Since the energy density of butanol is only 36 kJ/g, it is essential to minimize the energy consumed for butanol recovery in order to recover the highest net energy increase. The energy requirements for butanol recovery by conventional distillation, gas stripping, extraction, pervaporation, and adsorption-desorption have been estimated at 24, 21, 14, 9, and 8 kJ/g butanol, respectively [12,13], highlighting the superiority of adsorption-desorption with respect to energy return. An assessment conducted by Oudshoorn and his co-workers also demonstrated that adsorption- and pervaporation-based techniques are the most attractive recovery options for the recovery of butanol from aqueous solution [11]. Silicalite, bonopore, polyvinylpyridine, zeolite, activated carbon, and polymeric resins have been exploited for butanol adsorption. Being solid phase, the adsorbents have the advantages of complete immiscibility and ease of regeneration and reuse [13]. However, application of adsorbents in butanol fermentation has been limited by their low adsorption selectivity and capacity [12].

In our preliminary work, *Clostridium acetobutylicum* (*C. acetobutylicum*) cells were immobilized as a biofilm on a fibrous matrix, which increased the butanol tolerance and dramatically improved the ABE productivity of the cells [14]. A novel macroporous resin, KA-I, was also synthesized and used for the recovery of butanol from the model solution, with high selectivity and capacity [15,16]. Based on these results, we aimed to develop an integrated process to maximize the butanol production efficiency with minimal resource consumption. First, the adsorption behavior of KA-I during fermentation was investigated and controlled. Second, a system integrating a biofilm reactor with fixed-bed adsorption using KA-I was constructed and optimized for the production of ABE and acetoin with simultaneous product recovery.

## Results

### *In situ* product removal using the macroporous resin KA-I

#### Effects of resin amount

The biocompatibility of the KA-I resin and the effects of the resin on the metabolism of the cells were first investigated. Various amounts of resin (40 to 80 g/L) were directly added to planktonic cultures. The fermentation times were shortened from 80 h to between 45 and 70 h depending on the resin amounts (Table 1). When the fermentation was ended, the adsorbed solvents were eluted with methanol and quantified (see Methods). The solvent adsorption capacities of the resin were calculated as solvents adsorbed (mg) divided by the amount of resin (g) used in the fermentation. The KA-I resin had the highest adsorption capacity for butanol (maximum: 110 mg/g, obtained when 40 g/L resin was used), followed by adsorption of acetone. In contrast, no ethanol, acetic acid, acetoin, or glucose were detected in the eluent, indicating KA-I exhibited no adsorption capacity for these compounds at the end of the fermentation, which was consistent with the previous study using model solutions [15]. However, relatively high amounts of butyric acid were also adsorbed by the KA-I resin. Butyric acid and acetic acid were predominantly generated during the exponential growth of *C. acetobutylicum* and reached peak concentrations before being re-assimilated for ABE production. Adsorption of butyric acid led to an increased residual concentration, reducing the solvent yield. The greater the amount of resin added to the culture, the higher the residual levels of acids (including acetate) at the end of the fermentation. When an excessive amount (80 g/L) of resin was initially added to the culture, 5.46 g/L acids remained in the culture, which was 117% and 475% higher than the concentration of acids obtained after the addition of 50 g/L resin and in the control, respectively.

**Table 1:** Effects of resin quantity on the final product yield

**Resin**		**Butanol (g/L)**			**Butyric acid (g/L)**			**Acetic acid (g/L)**	**Acetoin (g/L)**	**Acetone (g/L)**
**Amount (g/L)**	**Time**^**a **^**(h)**	**Aqueous**	**Adsorbed**	**Total**	**Aqueous**	**Adsorbed**	**Total**			
0	0 to 80	11.8		11.8			0.29	0.66	1.81	4.63
40	0 to 70	6.87	4.42	11.3	0.50	0.37	0.87	0.81	2.22	3.82
50	0 to 60	6.25	4.76	11.0	0.77	0.46	1.23	1.28	3.61	2.02
	40 to 65	6.42	5.09	11.5	0.35	0.29	0.64	0.79	3.44	2.58
60	0 to 45	5.11	4.62	9.73	0.98	0.94	1.93	1.51	3.83	1.90
	40 to 60	5.55	4.81	10.4	0.58	0.54	1.12	0.77	3.20	2.92
80	0 to 45	2.51	5.31	7.81	1.26	2.27	3.53	1.93	3.09	1.78

^a^From the time the resin was added to the time the fermentation ended. For example, 40 to 65 means that resin was added to the culture at 40 h and the fermentation ended at 65 h. Data for ethanol are not shown. The experiments were performed in triplicate, and the mean value was calculated.

The amounts of residual acids could be reduced by adding the resin at a later time point. An optimal time point for adding the resin was found to be 40 h, when the acids started to be re-assimilated. Addition of 50 g/L of resin at 40 h, rather than at 0 h, led to a 43% reduction in the residual acids, and thus a higher butanol production was achieved. Based on the solvent production and fermentation time, 50 g/L was considered the optimal amount of resin to be added at 40 h.

#### Inverse relationship between the production of acetoin and acetone

An inverse relationship between acetoin and acetone production was observed in the adsorptive fermentation. Addition of resin significantly increased acetoin production and decreased acetone production (Table 1). An increase in the resin amount or addition at an earlier time led to a higher acetoin production and lower acetone production. When 60 g/L resin was initially added to the culture, acetoin production was as high as 3.8 g/L, whereas acetone production was only 1.9 g/L. Interestingly, an inverse relationship between acetoin and acetone production was also observed in the study by Doremus *et al*., who investigated the effects of pressure and agitation on ABE fermentation [17]. Thus, it appears that acetoin can be overproduced at the expense of acetone without negatively affecting butanol production. However, acetoin production by *C. acetobutylicum*, as reported in early studies, was usually not higher than 1.0 g/L [18]. To the best of our knowledge, 3.8 g/L is the highest yield of acetoin produced by *C. acetobutylicum.*

#### Fermentation kinetics

**Figure 1:**
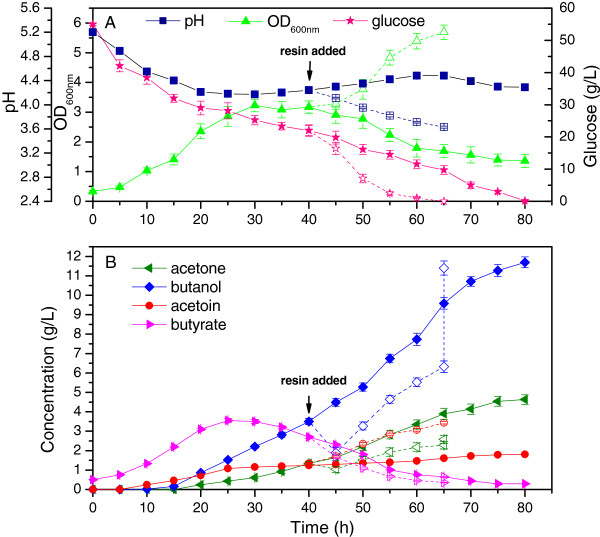
**Kinetics of batch fermentation by planktonic culture. (A)** pH, optical density (OD)_600nm_ and glucose consumption; **(B)** product concentrations during the fermentation process. Initially, experiments were performed in six Duran bottles under the same condition. At 40 h, three of the bottles were supplemented with KA-I resin (final concentration 50 g/L) (open symbol and dashed line), with the other ones left as control (solid symbol and solid line). The mean value (± SD) was calculated from the results of parallel runs. The vertical dashed lines represent the equivalent concentrations adsorbed by the resin (determined by dividing the adsorbed amounts by the volume of the fermentation broth) at the end of the fermentation.

As shown in Figure 1A, when 50 g/L resin was added to the culture at 40 h, the rates of cell growth and glucose consumption were significantly accelerated. Glucose was completely consumed in less than 65 h, with higher butanol productivity (0.30 g · L^-1^ · h^-1^) than in the control (0.25 g · L^-1^ · h^-1^). The aqueous concentrations of butanol and butyric acid decreased quickly (Figure 1B), and optical density (OD)_600 nm_ increased by 80.4% (from 3.17 to 5.72), instead of continuing to decline as observed in the control. Thus, it can be concluded that additional butyric acid and acetic acid were produced during the stimulated growth. The pH decreased throughout this period to 3.63, a low value that was not observed in the control. Despite the low pH value, the *C. acetobutylicum* cells still exhibited efficient fermentation, suggesting that butyric acid and butanol exerted more dominant effects on *C. acetobutylicum* than other acids affecting pH. In conclusion, *in situ* extractive fermentation with KA-I resin improved the cell growth and accelerated the fermentation process, indicating good biocompatibility of the KA-I resin.

### Batch fermentation in biofilm reactors coupled with fixed-bed adsorption

#### Biofilm reactor

**Figure 2:**
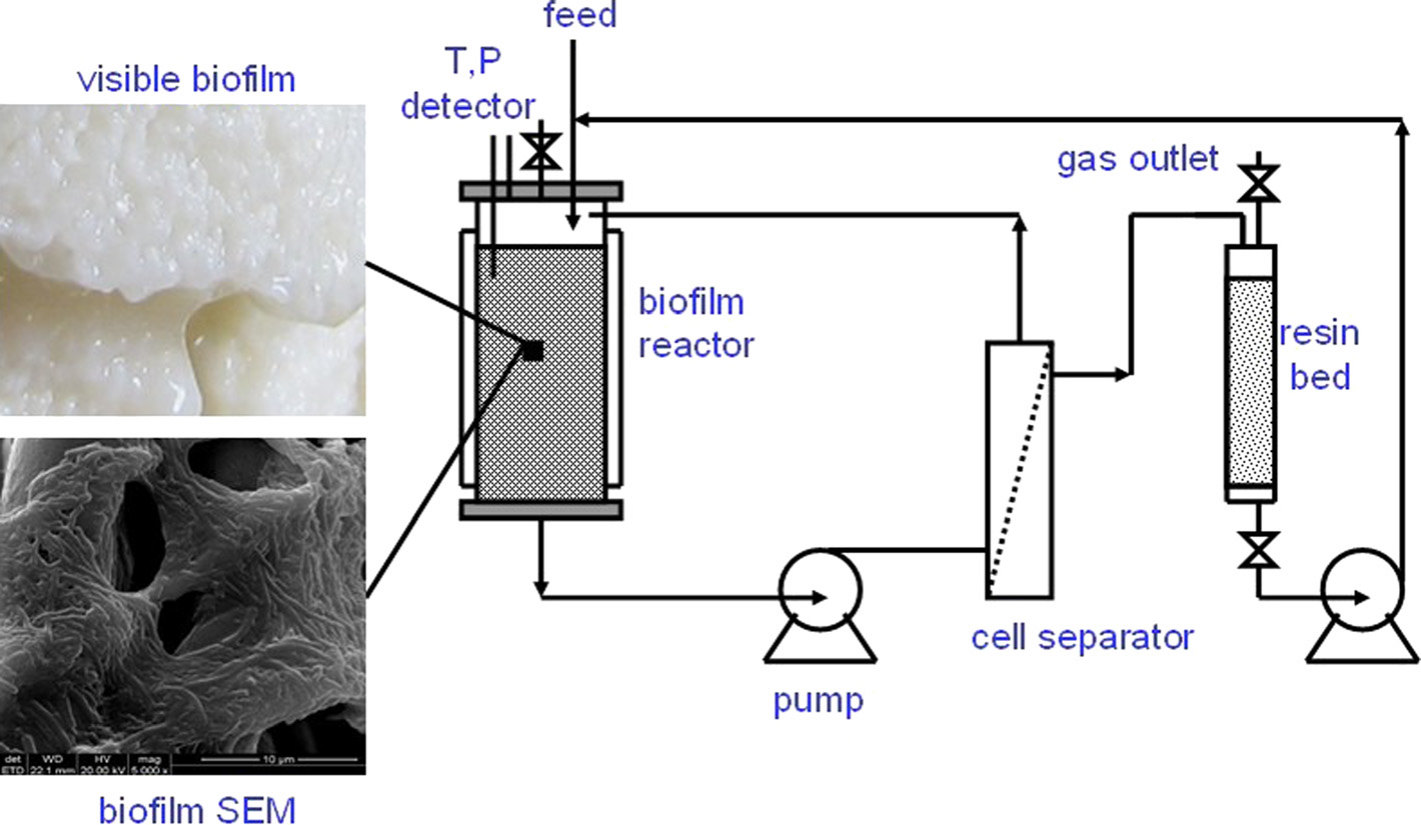
**Schematic diagram of a biofilm reactor coupled to fixed-bed adsorption.** The KA-I resin was packed in glass columns of 250-mm length and 30-mm diameter. Approximately 50 to 60 g of the resin was loaded in each column. After autoclaving, the resin column was connected to a 2-L biofilm reactor to construct an online product removal system. With feeding of concentrated P2 medium, fed-batch fermentation can be performed. Operation details are described in the Methods section. SEM, scanning electron microscopy; T, temperature; P, pressure.

Different materials such as resins, activated carbon, silk, cotton, or polyester, were used to support biofilm formation. Generally, *C. acetobutylicum* B3 biofilms on these carriers were visible after 48 h in culture, suggesting a good ability of *C. acetobutylicum* B3 cells to form a biofilm on solid surfaces. However, fermentation rates and product patterns of the biofilms differed from each other (data not shown). In the present study, a fibrous matrix (cotton towel, Skyshow Textiles Co. Ltd. Shanghai, China) was selected after experimental examinations as the support for *C. acetobutylicum* B3 biofilm formation. A thick, sticky layer of biofilm stacked on the matrix surface was observed. Scanning electron microscopy (SEM) revealed that the cells formed aggregates and were effectively immobilized by the extracellular polymeric substance that they produced (Figure 2). The performance of the biofilm reactors is summarized in Table 2. Batch fermentations with a stable biofilm reactor could be completed in approximately 12 h, with a 5-fold higher productivity compared with a planktonic cell reactor. Furthermore, the cell density of the bulk medium in the biofilm reactors was about 10-fold lower than that of the planktonic cell bioreactors, indicating that the cells preferred to propagate on the fibrous matrix rather than in the bulk medium. While there have been many efforts to increase the cell density in a traditional planktonic cell reactor to achieve an increased production rate [19,20], in the present study, a minimum turbidity of the bulk medium in the biofilm reactors is desired to lower the burden of the cell separator. Apparently, this low cell-density was beneficial for the long runtime of the integrated fermentation process. Furthermore, in the biofilm reactor, fermentations can be performed in a repeated batch mode or a continuous fermentation mode. Interestingly, contrary to the substitution of acetone by acetoin in the *in situ* adsorption fermentations, production of acetoin in the biofilm reactors was much lower, whereas the production of acetone was higher.

**Table 2:** Performance of the biofilm reactors with butanol maintained at different levels

**Fermentation process**	**Ethanol(g/L)**	**Butanol(g/L)**	**Acetone(g/L)**	**Acetoin(g/L)**	**Butyrate(g/L)**	**Yield(g/g)**	**Productivity(g · L**^**-1**^ **· h**^**-1**^**)**	**MaximumOD**_**600 nm**_
Biofilm reactor, fixed-bed adsorption 2.5 to 3.2 g/L butanol	1.37	10.6	5.73	1.51	1.88	0.32	1.47	7.88
Biofilm reactor, fixed-bed adsorption 4.0 to 5.5 g/L butanol	1.44	11.1	6.32	1.23	0.95	0.33	1.60	3.46
Biofilm reactor, fixed-bed adsorption 5.2 to 6.4 g/L butanol	1.49	11.7	6.43	1.25	0.49	0.35	1.53	1.55
Biofilm reactor, without adsorption	1.48	11.6	6.70	0.98	0.17	0.35	1.66	0.30
Traditional planktonic cell fermentation, without adsorption	2.1	11.8	4.6	1.8	0.29	0.34	0.25	3.2

The data represent the averages of two independent fermentations. Data for acetate are not shown. Typically, acetate displayed a change trend similar to that of butyrate. OD, optical density.

#### Importance of the butanol level

The online fixed-bed adsorption system was constructed by packing the KA-I resin in glass columns and connecting the glass columns to the biofilm reactor (Figure 2). The butanol titer was maintained at different levels to investigate the effects of the titer on the fermentation. As shown in Table 2, lowered butanol titers in the biofilm reactor did not improve the productivity but increased the cell density of the bulk medium as well as the residual butyric acid level at the end of the fermentation, resulting in a low butanol yield. In addition, low butanol titers also significantly decreased the adsorption capacity of the KA-I resin (data not shown). Hence, the butanol titer was maintained above 6 g/L in the subsequent experiments.

### Fed-batch fermentation in biofilm reactors coupled with fixed-bed adsorption

#### Fermentation kinetics

**Figure 3:**
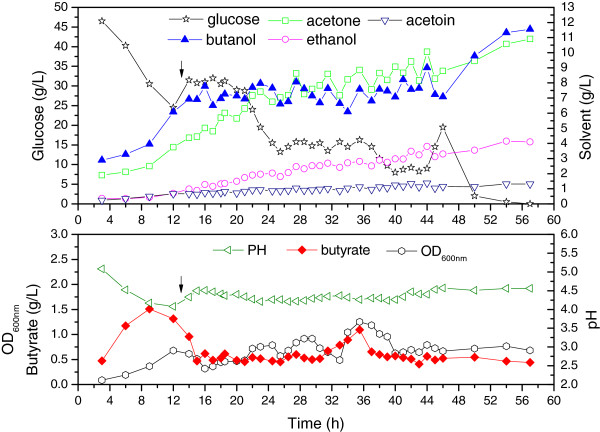
**Kinetics of fed-batch fermentation in the biofilm reactor with fixed-bed adsorption.** After 13 h of batch culture, concentrated medium was fed into the reactor to maintain desired glucose levels and the fixed-bed adsorption was started, as is indicated by arrows. Butanol was selectively adsorbed by the KA-I resin, whereas other solvents accumulated in the fermentation broth. OD, optical density.

As shown in Figure 3, after 13 h of batch culture, concentrated P2 medium (500 g/L glucose) was fed into the reactor via a peristaltic pump. Glucose was fist maintained at approximately 30 g/L for 10 h and then at approximately 15 g/L for 10 h to investigate its effect on fermentation. Whenever the glucose concentrations in the reactor fell below the pre-determined levels, the feed pump was turned on at a flow rate of 15 mL/h until the predetermined levels were reached. The butanol titer in the fermentation broth was also maintained between 6.5 and 8.5 g/L with fixed-bed adsorption. Acetone, ethanol, and acetoin gradually accumulated owing to the selective adsorption of the KA-I resin. In batch fermentations, low glucose concentrations led to increased residual acids [21]. However, in the fed-batch mode, under a controlled butanol titer, 15 and 30 g/L glucose did not significantly affect the acid production or the cell density in the medium. Both the level of butyrate and the OD_600nm_ were relatively constant, except for a sudden increase observed when the butanol level was below 6.5 g/L (Figure 3). Hence, it was important to maintain butanol at a higher level to reduce the butyrate level and the cell density. The pH was also constant at 4.2, in contrast to low value of 3.6 observed in the *in situ* adsorption experiment. The fermentation was stopped when the residual glucose was completely used owing to depletion of the resin columns. The results obtained are summarized in Table 3. An equivalent solvent concentration of 59.8 g/L was achieved, which was about three times higher than that obtained in batch fermentation.

**Table 3:** Results of fed-batch fermentation in biofilm reactors coupled with fixed-bed adsorption

	**Fermentation with selective adsorption of butanol**	**Fermentation with co-adsorption of acetone**	**Fermentation with redox modulation**
Fermentation time (h)	57	64	135
Glucose fermented (g/L)	198	297	367
Resin used (g)	418	812	910
Adsorbed ethanol (g)	1.8	0.82	2.2
Adsorbed acetone (g)	12.3	35.7	23.6
Adsorbed butanol (g)	42.1	68.1	84.7
Adsorbed acetoin (g)	0.40	2.4	1.2
Adsorbed butyrate (g)	1.9	3.1	8.7
Equivalent ethanol concentration (g/L)	5.1	4.8	6.8
Equivalent acetone concentration (g/L)	14.8	30.9	28.8
Equivalent butanol concentration (g/L)	38.2	58.3	92.6
Equivalent acetoin concentration (g/L)	1.7	2.6	2.6
Equivalent butyrate concentration (g/L)	1.7	2.4	10.8
^a^Solvent concentration (g/L)	59.8	96.5	130.7
^a^Solvent productivity (g · L^-1^ · h^-1^)	1.05	1.51	0.97
^a^Solvent yield (g/g)	0.302	0.325	0.356
^b^Total product yield (g/g)	0.311	0.333	0.386
Butanol-acetone molar ratio	2.6^c^	1.9	3.2

^a^Solvent: ABE and acetoin. ^b^Total product: ABE, acetoin, and butyrate. Acetate is not considered to be a product. ^c^The increased butanol-acetone ratio was probably due to the evaporation of acetone.

#### Kinetics of fixed-bed adsorption

**Figure 4:**
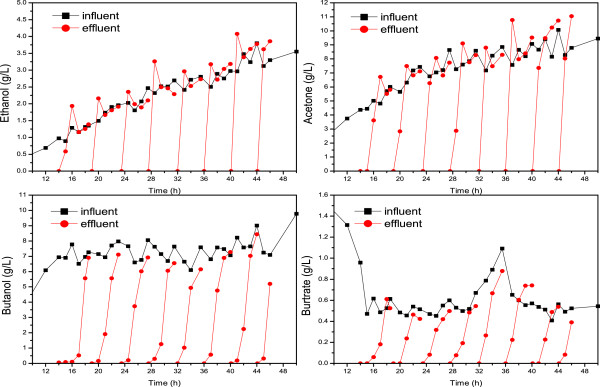
**Kinetics of the fixed-bed adsorption.** The fixed-bed adsorption was started at13 h. A sample before the adsorption (influent, black square) and a sample after the adsorption (effluent, red circle) were withdrawn simultaneously every time to determine the extent of resin saturation. A higher sorbate titer in the effluent meant that the sorbate initially adsorbed onto the KA-I resin was subsequently eluted in the butanol adsorption process. The resin column was replaced with a new resin column when saturated by butanol.

Figure 4 shows the dynamic process of the fixed-bed adsorption. Although the *in situ* adsorption experiments showed that the KA-I resin had no adsorption capacity for ethanol at the end of the batch fermentation, the fixed-bed adsorption curve revealed that initially, ethanol, acetone, and butanol could be simultaneously adsorbed by the KA-I. However, ethanol, followed by acetone, was subsequently eluted, whereas butanol continued to be adsorbed, which indicated that ethanol was a weakly retained component and butanol was a strongly retained component; acetone retention was intermediate. Similar to the *in situ* adsorption experiments, butyrate was also significantly adsorbed by the fixed-bed columns, and it appeared that butyrate could not be competitively eluted by butanol. The KA-I resin, with a cross-linked polystyrene framework, is a weakly polar resin. The adsorption affinity for ABE showed a correlation with the polarity from the sorbate, that is, the affinity for the sorbate decreased with the increase in the sorbate polarity, from butanol to acetone to ethanol [22]. However, the adsorption behavior of butyrate was not in line with this. The high affinity for butyrate might be attributed to the hydrophobic interactions between the alkyl chain of butyrate and the aromatic groups of the KA-I resin [15].

#### Fermentation with co-adsorption of acetone

**Figure 5:**
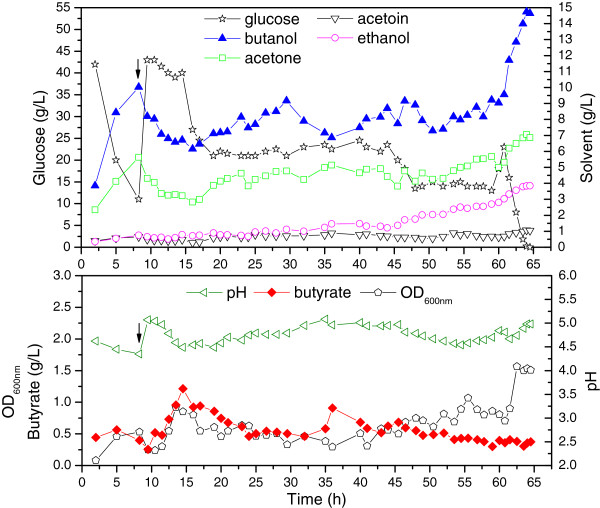
**Kinetics of fed-batch fermentation in the biofilm reactor with co-adsorption of acetone.** After 8 h of batch culture, concentrated medium was fed into the reactor to maintain desired glucose levels and the fixed-bed adsorption was started, as is indicated by arrows. By co-adsorption of acetone using KA-I resin, the acetone titer in the fermentation broth was also maintained at a relatively stable level. OD, optical density.

In the fermentation process by *C. acetobutylicum*, acetone was not as toxic as butanol. However, selective adsorption of butanol by the KA-I resin could make acetone a predominant inhibitory product in view of its quantity. More importantly, high acetone levels in the fermentation broth could result in serious mass loss of acetone due to its high volatility, leading to a low solvent yield (Table 3). Considering the competitive adsorption behavior of butanol with respect to acetone, as indicated by the fixed-bed adsorption dynamics, the resin columns were displaced properly before being saturated by butanol to enhance acetone adsorption and lower the level of acetone in the fermentation broth. As shown in Figure 5, butanol was maintained at a higher level (7 to 9 g/L) to further reduce the cell density and butyrate level in the medium. When the fixed-bed adsorption was stopped owing to the maintenance of the filtration unit, residual glucose was completely consumed, and the final aqueous butanol level reached 14.7 g/L. Three different levels (42, 23 and 13 g/L) of glucose were maintained during the fermentation to investigate the effect of glucose again. Similar to the previously observed results, the butanol titer rather than the glucose concentration, significantly affected butyrate production. With co-adsorption of acetone, the aqueous acetone level in the fermentation broth was successfully maintained at approximately 5 g/L. Although the adsorbed butanol level was reduced from 101 to 83.8 mg/g resin, the adsorbed acetone level was increased from 29.4 to 43.9 mg/g resin. The final equivalent solvent concentration was 96.5 g/L, which was approximately five times higher than that of the batch fermentation. The solvent yield was also partly recovered and thus higher than that of the fermentation with selective adsorption of butanol (Table 3).

#### Redox modulation for improved product yield

During ABE fermentation by *C. acetobutylicum*, the release of H_2_ causes a deficiency in nicotinamide adenine dinucleotide (phosphate) (NAD(P)H) reducing equivalents used for ethanol and butanol production. Therefore, a large quantity of oxidation products (especially acetate and acetone) is commonly produced, resulting in a low alcohol yield [14]. In fact, acetone was produced in greater quantities in the biofilm reactor than in the planktonic cell reactor due to the increased H_2_ release. Methyl viologen (MV) was used to enhance butanol production because MV can divert the electron flow from H_2_ to NAD(P)H [23]. The results are summarized in Table 3. With addition of 1 mM MV, butanol production was markedly increased whereas acetone was reduced. Thus, the butanol-acetone molar ratio increased from 1.9 to 3.2. More importantly, when one glucose molecule is converted to the C4 compound butanol instead of the C3 compound acetone, the mass yield is expected to increase. Indeed, the total solvent yield (ABE and acetoin) upon MV addition increased by 9%. Redox modulation by MV addition also stimulated butyrate production, consistent with a previous report [4]. The butyric acid was maintained at approximately 1.5 g/L throughout the fermentation, which was a much higher level than the average of 0.5 to 0.7 g/L observed when no MV was added. Due to the adsorption by the KA-I resin, the final butyrate concentration reached 10.8 g/L. Therefore, the total product yield (ABE, acetoin, and butyric acid) was further increased by 16%. This increased product yield will reduce carbon emission in the ABE fermentation. In addition, MV was not adsorbed by the KA-I resin, suggesting that MV could be reused in the fermentation. However, the use of MV resulted in a slower fermentation rate, likely due to the inhibition of cell growth [23] or through a toxic effect of butyric acid on metabolism [24] during the prolonged fermentation.

#### Comparison with other studies

Although adsorption of butanol from model solutions onto various resins has been extensively studied, there have been few reports of the application of resins for butanol fermentation, perhaps due to relatively low adsorption capacities of previously studied adsorbents (Table 4). In small-scale batch fermentations, poly(styrene-co-divinylbenzene)-derived materials were demonstrated to be excellent adsorbents for butanol with high adsorption capacity [12,25]. However, continuous or fed-batch fermentation with this resin has not yet been reported. Yang and Tsao used polyvinylpyridine as an adsorbent for ABE in repeated fed-batch fermentations [26]. The equivalent solvent concentration and the productivity obtained in this integrated process increased by 140% and 320%, respectively, compared with the solvent concentration and productivity obtained through traditional batch fermentation. In this study, fermentations with fixed-bed adsorption using KA-I were conducted in a biofilm reactor. The biofilm could maintain its metabolic activity during a long period of feeding fermentation; thereby, a high solvent concentration was achieved. The equivalent solvent concentrations and the productivities obtained in the fermentation were four to six times and three to five times higher, respectively, than those obtained by conventional fermentation. In the present study, by redox modulation using MV, a relatively high yield of solvent was also achieved. However, similar to many other resins, KA-I also showed a high adsorption affinity for butyrate, which could affect butanol yield. A genetically engineered strain with a modified product spectrum (for example, diminished acetone, acetate, and butyrate) may compensate for the imperfect selectivity of the adsorbents and make adsorption more competitive than other recovery methods.

**Table 4:** Performance of different types of resin used as adsorbents during butanol fermentation

**Fermentation**	**Resin**			**Performance**			**Reference**
	**Type**	**Selectivity**	**Capacity**^**a**^**(mg/g)**	**Equivalent concentration(g/L)**	**Solvent productivity(g · L**^**-1**^ **· h**^**-1**^**)**	**Solvent yield(g/g)**	
Batch fermentation, *in situ* adsorption	XAD-8	Mainly butanol, isopropanol, glucose	30	12.6	Approx. 0.13	-	[27]
Two-stage continuous fermentation, in-line adsorption	XAD-16	Butanol, acetone, acetate, and butyrate; no lactose	64	11.01	Approx. 1.5	0.30 to 0.36	[28]
Repeated batch fermentation, batch adsorption	Bonopore, copolymers of divinylbenzene, and styrene	Solvent and acids	43	-	-	-	[29]
Batch fermentation,* in situ* adsorption	Dowex Optipore SD-2ploy(styrene-co-DVB) derived	Mainly butanol and butyrate	264	Approx. 37	Approx. 0.51	0.27 to 0.40	[12]
Repeated fed-batch fermentation, fixed-bed adsorption	Reillex 425, polyvinylpyridine	ABE and acids; no glucose	Approx. 61	47.2	1.69	0.32	[26]
Fed-batch fermentation, fixed-bed adsorption	KA-I, cross-linked polystyrene framework	Butanol, butyrate, and acetone; no glucose	84	96.5	1.51	0.33	This work
Fed-batch fermentation, fixed-bed adsorption, MV addition	KA-I, cross-linked polystyrene framework	Butanol, butyrate, and acetone; no glucose or MV	93	130.7	0.97	0.36	This work

^a^Calculated as butanol adsorbed (mg) divided by the amount of resin (g) used in the fermentation process. MV, methyl viologen; ABE, acetone-butanol-ethanol; approx., approximately.

## Discussion

### Acetoin: a value-added product

Interestingly, an inverse relationship was observed between the production of acetoin and acetone. Acetoin (3-hydroxybutanone) is a fine chemical widely used for food flavoring and as a fragrance. Acetoin is also used to synthesize novel optically active α-hydroxyketone derivatives and liquid crystal composites [30]. Recently, the ratio of d-acetoin to l-acetoin produced by *Clostridium* strain ATCC 824 was found to be 12:1 [31]. In contrast, acetoin produced by butanediol-producing strains are often racemic. The inverse relationship suggests that ABE fermentation may be switched to acetoin-butanol-ethanol fermentation, which is a promising approach to improve the product value of ABE fermentation. It was presumed that acetoin is a structural analog of acetoacetate and could not be decarboxylated by acetoacetate decarboxylase, which would impede acetone production [17]. However, exogenous addition of 1, 2, or 4 g/L acetoin to the fermentation broth failed to lower the acetone production (data not shown). Recent insights from molecular studies indicate that acetoin is the product of *als*SD (encoding an α-acetolactate synthase and an acetolactate decarboxylase), which is regulated by the transcription factor *als*R in response to acetate accumulation and a pH change in *Bacillus subtilis*[32]. Study of disruption of *als*R in *C. acetobutylicum* is ongoing to better understand the mechanisms underlying the inverse relationship.

### High productivity of the biofilm reactor

Our previous study demonstrated that *C. acetobutylicum* cells exhibited improved butanol tolerance and production rate in biofilm compared with planktonic cells [14]. Thus, the *C. acetobutylicum* B3 biofilm was used for butanol fermentation in this study. The solvent production rate was increased dramatically, from 0.25 to 0.30 to 0.97 to 1.66 g · L^-1^ · h^-1^. Meanwhile, in the biofilm reactor, fermentation can be performed in a repeated batch mode or a continuous mode, which can overcome the drawbacks of batch operation, such as down times and long lag periods. Thus, the reactor productivities were greatly improved, which can significantly reduce capital investment and operational costs [2]. Furthermore, the cells can remain in the biofilm during a prolonged feeding fermentation or repeated batch fermentations, and the already established solvent-producing capacity of the cells would increase the carbon flux to solvent production rather than biomass synthesis.

### Conservation of energy and water by simultaneous product removal

Due to product toxicity, *C. acetobutylicum* fermentation rarely produces >13 g/L butanol [4]. Product recovery from the dilute fermentation broth by conventional distillation is energy intensive because water is the major component, with a boiling point below that of butanol (100 versus 117°C). When the fermentation was coupled to a simultaneous adsorption process using KA-I, the product was removed, and the levels of butanol and acetone were maintained below the inhibitory threshold. Thus, the fermentation could be prolonged with a continuous feed of highly concentrated nutrients, and a high equivalent-solvent titer was achieved. When the resin columns were eluted with methanol, the average concentration of butanol in the eluent could be increased by approximately 6-fold to 50 to 60 g/L. Distillation technology showed that if the butanol concentration could be raised from 12 to 19 g/L, the energy consumption of butanol recovery from the fermentation broth could be cut in half [16]. Together with the low methanol boiling point (64°C), the high butanol titer in methanol would significantly reduce the energy requirement for butanol separation. Moreover, the high equivalent solvent concentration obtained in the integrated process meant that the fermentation was performed with a highly concentrated medium, which reduced the water usage and resulted in significant savings in sterilization and wastewater treatment.

### Improvement of yield by redox modulation

Theoretically, butanol can be produced from glucose with 100% molar yield. However, release of H_2_ causes a deficiency of the reducing equivalents NAD(P)H, thus resulting in a large quantity of oxidation products, especially acetone [14]. Except for acetoin, butanol is the preferred product, attracting the highest price of the solvents. Addition of MV diverted the electron flow from H_2_ to NAD(P)H [23], which facilitated acetoacetyl-CoA reduction to butanol rather than decarboxylation to acetone. Thus, the butanol-acetone ratio was improved, and the emission of H_2_ and CO_2_ was reduced, leading to increased product yield. Hence, redox modulation of *C. acetobutylicum* metabolism by small-molecule effectors can be used to improve both the butanol-acetone ratio and the total solvent yield in ABE fermentation, which has been rarely achieved by genetic manipulations [4,33,34].

In summary, the process developed in this study could potentially (1) reduce capital investment and operational costs due to high reactor productivity, (2) reduce energy consumption and wastewater treatment due to the highly concentrated medium and high solvent titer, and (3) increase product value and reduce gas emission due to the improved butanol ratio and solvent yield. Genetically engineered strains with modified product spectra (for example, diminished acetone and acid production, or selective butanol production) will make this process more economically favorable.

## Conclusions

KA-I resin can be used as a good adsorbent for product recovery in ABE fermentation. Use of KA-I resin enhanced acetoin production at the expense of acetone production during the fermentation. To reduce butyrate adsorption by KA-I, the butanol titer should be maintained at a relatively high level (>6.5 g/L). The biofilm reactor dramatically enhanced the fermentation productivity compared with the planktonic reactor. In fed-batch production with simultaneous product recovery, the equivalent solvent concentration was much higher than in a conventional fermentation. Redox modulation was effective for improving the butanol-acetone ratio and the total product yield. While molecular technologies focusing on strain construction are becoming mainstream in the development of economically viable biobutanol production, process technologies should not be overlooked because they can be an effective means of fully exploiting the productivity of a strain and maximizing the production efficiency.

## Methods

### Organism and culture conditions

*C. acetobutylicum* B3 (CGMCC number 5234) was used in all of the fermentation experiments. The strain was stored in 30% (v/v) glycerol at −80°C and was grown in solid reinforced clostridia medium (RCM) at 37°C in an anaerobic chamber (Bug Box, Ruskinn Technology, Leeds, UK). Cultures of *C. acetobutylicum* B3 were grown in modified P2 medium (containing 10 g/L glucose as the sole carbohydrate source) for seed culture. Unless otherwise stated, the fermentation experiments were performed in P2 medium (comprising glucose, 60 g/L; K_2_HPO_4_, 0.5 g/L; KH_2_PO_4_, 0.5 g/L; CH_3_COONH_4_, 2.2 g/L; MgSO_4_ · 7H_2_O, 0.2 g/L; MnSO_4_ · H_2_O, 0.01 g/L; NaCl, 0.01 g/L; FeSO_4_ · 7H_2_O, 0.01 g/L; *p*-aminobenzoic acid, 1 mg/L; thiamine, 1 mg/L; and biotin, 0.01 mg/L) [35] at 37°C with 10% inoculum (v/v).

### Biofilm formation and repeated batch fermentations

For biofilm formation, a 2-L stainless steel column was packed with a spiral fibrous matrix (90 g). The columns were sterilized at 121°C for 30 minutes before use. Subsequently, 150 mL of 12-h-old seed culture was inoculated into 1.5 L of P2 medium and allowed to grow statically for 20 h at 37°C. Next, the culture was transferred to the stainless steel column and circulated through the fibrous matrix at a pumping rate of 25 mL/minute to allow the cells to immobilize onto the fibrous matrix and form a biofilm. The temperature was controlled via connection of the jacketed column to a warm water bath at 37°C. After 20 h of continuous circulation, the broth was replaced with fresh P2 medium to start the ABE fermentation. The medium circulation rate was maintained at 35 mL/minute via a peristaltic pump during fermentation. When a batch ended, fresh P2 medium was fed to the column to replace the fermentation broth. Subsequently, the medium was circulated again, and the next batch fermentation was started with the biofilm on the fibrous matrix.

### Scanning electron microscopy

For SEM, the cells were collected at 48 h. A piece of cotton towel was harvested and rinsed twice with PBS buffer (137 mM NaCl, 2.7 mM KCl, 8 mM Na_2_HPO_4_, and 2 mM KH_2_ PO_4_, pH 7.40) to remove contaminating planktonic cells before being freeze-dried using the FreeZone Freeze Dry System (Labconco Corporation, Kansas City, MO, USA). The samples were scanned and photographed by SEM (Philips XL-30 ESEM, Eindhoven, Holland).

### *In situ* product adsorption

*In situ* adsorption experiments were conducted in 500-mL Duran bottles with a 300-mL working volume. The macroporous adsorption resin KA-I, with a cross-linked polystyrene framework, was supplied by the National Engineering Technique Research Center for Biotechnology (Nanjing, China) [15]. The resin was first soaked in methanol for 24 h and then washed with water before being pumped dry and weighed. Subsequently, the resin was sterilized in a small amount of deionized water by autoclaving for 15 minutes at 121°C. The desired mass of resin was added to 300 mL of culture medium at different times according to the experimental design.

### Fixed-bed adsorption

A schematic diagram of the process is shown in Figure 2. When the butanol titer reached the predetermined threshold, the fermentation broth was transferred to a microporous filtration unit (Tianjin MOTIMO Membrane Technology Co., Model MOF-1b, Tianjin, China) to separate the cells before being loaded onto the resin bed. The effluent was transferred back to the reactor for further fermentation. In the fed-batch mode, 1 L of P2 medium was initially fed into the reactor. Subsequently, P2 medium containing a high glucose concentration (500 g/L) was fed into the reactor using a peristaltic pump to maintain the glucose concentration within the desired range. When the resin column was saturated, it was replaced with a new resin column.

### Redox modulation

MV was purchased from Sigma-Aldrich (Saint Louis, Missouri, USA ) as the dichloride hydrate. It was prepared as a sterile stock solution and added to the fermentation broth as necessary to achieve a final concentration of 1 mM. The MV concentration was determined spectrophotometrically as described previously [36] to determine whether it had adsorbed to the KA-I resin.

### Solvent desorption and resin regeneration

The resin in the *in situ* adsorption experiment was collected at the end of the fermentation and washed gently with 100 mL of deionized water. Subsequently, the resin was soaked in 500 mL of methanol and agitated at 200 rpm for 30 minutes at 25°C. The supernatant liquids were filtered and analyzed by gas chromatography. The solvents adsorbed to the resin packed in the glass columns were desorbed in a fixed-bed mode using one bed volume of 85% (v/v) methanol. The solvent recovery from the KA-I resin was >99%. The resin could be regenerated using two bed volumes of water (for details, see [15]).

### Metabolite analyses and calculations

The cell concentration was determined spectrophotometrically as the OD_600nm_. The concentrations of glucose, acetate, and other organic acids were determined by HPLC analysis (Agilent 1100 series, Hewlett-Packard, California, USA), using an Aminex HPX-87H ion exclusion column (300 × 7.8 mm; Bio-Rad Laboratories, Hercules, CA, USA) heated to 50°C. The analytes were separated with a mobile phase of 5.0 mM H_2_SO_4_ at 0.6 mL/minute and detected using a refractive index detector. Acetone, ethanol, butanol, butyric acid, and acetoin were analyzed using gas chromatography (7890A, Agilent, Wilmington, DE, USA) equipped with a flame ionization detector and an Agilent HP-INNOWAX column (0.25 mm × 60 m).

The equivalent solvent concentration was calculated as the total amount of solvent produced, including the aqueous volume and the adsorbed mass (g) divided by the total volume of medium (L) in the fermentor, cell separator, and samples. The solvent productivity was calculated as the equivalent solvent concentration (g/L) divided by the fermentation time (h). The yield was calculated as the total mass of solvent (ABE and acetoin) produced (g) divided by the total mass of glucose utilized (g).

## Abbreviations

ABE: Acetone-butanol-ethanol; HPLC: High performance liquid chromatography; MV: Methyl viologen; NAD(P)H: Reduced nicotinamide adenine dinucleotide (phosphate); NTG: Nitrosoguanidine; OD: Optical density; PBS: Phosphate-buffered saline; SEM: Scanning electron microscopy.

## Competing interests

The authors have declared that no competing interests exist.

## Authors’ contributions

DL, YC, JW, and HY designed experiments. DL, FD, TZ, and HR performed experiments. BL, HN, ZC, XL, JX, and XC contributed materials and sample analysis. DL and YC analyzed data. DL and HY wrote the manuscript. All authors have read and approved the final manuscript.
